# Hypoxia affects Slc7a5 expression through HIF‐2α in differentiated neuronal cells

**DOI:** 10.1002/2211-5463.12559

**Published:** 2019-01-07

**Authors:** Yuki Onishi, Manami Hiraiwa, Hikari Kamada, Takashi Iezaki, Takanori Yamada, Katsuyuki Kaneda, Eiichi Hinoi

**Affiliations:** ^1^ Laboratory of Molecular Pharmacology Division of Pharmaceutical Sciences Kanazawa University Graduate School Japan; ^2^ Venture Business Laboratory Organization of Frontier Science and Innovation Kanazawa University Japan

**Keywords:** amino acid transporter, branched‐chain amino acid, Hif‐2α, hypoxia, neuron, Slc7a5

## Abstract

An imbalance of branched‐chain amino acids (BCAAs) in the brain may result in neuropathological conditions, such as autism spectrum disorders. The L‐type amino acid transporter 1 (LAT1), encoded by the solute carrier transporter 7a5 (*Slc7a5*) gene, is critical for maintaining normal levels of BCAAs in the brain. However, our understanding of the mechanisms that regulate the expression of LAT1/*Slc7a5* in neurons is currently limited. Here, we demonstrate that hypoxic conditions result in upregulated expression of *Slc7a5* in differentiated neuronal cells (Neuro2A cells induced to differentiate using all‐*trans* retinoic acid). Mechanistically, hypoxia‐induced expression of *Slc7a5* is markedly reduced by short hairpin RNA (shRNA)‐mediated knockdown of hypoxia‐inducible factor 2α (HIF‐2α), but not by shRNA targeting HIF‐1α, in differentiated neuronal cells. Moreover, hypoxia increased the binding of HIF‐2α to the proximal promoter of *Slc7a5* in differentiated neuronal cells. These results indicate that hypoxia directly enhances the recruitment of HIF‐2α to the proximal promoter of *Slc7a5*, resulting in its upregulated expression in differentiated neuronal cells. These findings indicate that *Slc7a5* may be a novel gene responsive to hypoxia in a HIF‐2α‐dependent manner in differentiated neuronal cells.

**Abbreviations**
ASD: autism spectrum disorder
ATRA: all‐*trans* retinoic acid
BBB: blood–brain barrier
BCAA: branched‐chain amino acid
ChIP: chromatin immunoprecipitation
CNS: central nervous system
Glut1: glucose transporter‐1
HIF: hypoxia‐inducible factor
Hmox1: heme oxygenase‐1
HRE: HIF‐responsive element
LAT: L‐type amino acid transporter
mTORC1: mechanistic target of rapamycin complex 1
qPCR: quantitative PCR
shRNA: short hairpin RNA
Slc7a5: solute carrier transporter 7a5

It is well recognized that amino acids are capable of regulating cellular function through the modulation of signaling pathways (e.g., the mechanistic target of rapamycin complex 1 [mTORC1]) in various cell types, including the main cell types characterizing the central nervous system (CNS), neurons, and glial cells 1, 2, 3. Moreover, branched‐chain amino acids (BCAAs) serve as precursors for the synthesis of neurotransmitter glutamate. Pathologically high levels of BCAAs, as observed in maple syrup urine disease, cause excessive glutamatergic signaling and neurological symptoms, such as cognitive dysfunctions, seizures, and hypotonia 4, 5. Moreover, it has been reported that mutations in the gene encoding branched‐chain keto acid dehydrogenase kinase, the rate‐limiting enzyme in the catabolic pathway of BCAAs, result in autism spectrum disorder (ASD) and fine motor coordination problems because of abnormally low levels of BCAAs in the serum and brain 6.

Because a constant supply of BCAAs from the periphery is essential for the functions of the brain during development and its homeostasis, factors facilitating the uptake of BCAA may be critical for the proper functioning of the CNS. The L‐type amino acid transporter 1 (LAT1), encoded by the solute carrier transporter 7a5 (*Slc7a5*) gene, forms a heterodimer with a type II glycoprotein, the heavy chain of the 4F2 cell‐surface antigen (4F2hc/CD98), via a disulfide bond 7, 8. It has been shown that LAT1 is expressed in the blood–brain barrier (BBB) for maintaining normal levels of BCAAs in the brain. Moreover, LAT1 deficiency in cerebral endothelial cells causes neurological and behavioral abnormalities associated with ASD 9, 10, 11. Although the pivotal expression and crucial role of *Slc7a5* in the BBB (particularly in endothelial cells) have been determined, there is limited evidence regarding the potential expression of *Slc7a5* and molecular mechanisms underlying the regulation of its expression in neuronal cells under pathological conditions.

Hypoxia‐inducible factors (HIFs) are transcription factors that transcriptionally regulate a large number of genes involved in angiogenesis, glycogenesis, and cell survival, promoting adaptation to hypoxic/ischemic stress in response to low tissue oxygenation during ischemic stroke 12, 13. HIFs are heterodimers, comprising one of the three major oxygen labile HIF‐α subunits (HIF‐1α, HIF‐2α, or HIF‐3α) and a constitutive HIF‐1β subunit. Among these, the HIF‐1α/HIF‐1β and HIF‐2α/HIF‐1β dimers are the primary factors regulating hypoxic transcriptional responses in most mammalian cells 14.

In this study, we investigated whether *Slc7a5* is regulated by hypoxia to mimic an ischemic stroke environment in neuronal cells, and the molecular mechanisms underlying the regulation of the expression of *Slc7a5*.

## Materials and methods

### Culture of Neuro2A cells

Neuronal Neuro2A cells derived from mouse neuroblastoma exhibited properties of neuronal progenitor cells, committing to neuron‐like cells in the presence of all‐*trans* retinoic acid (ATRA). Undifferentiated Neuro2A cells were cultured in Dulbecco's modified Eagle's medium (DMEM) supplemented with 10% FBS. Neuro2A cells were inoculated in culture wells at a density of 10^4^ cells·mL^−1^ in DMEM supplemented with 10% FBS for 24 h, followed by medium change to DMEM supplemented with 2% FBS and 20 μm ATRA for differentiation, as previously described 15.

Cells were cultured in dishes placed in a jar containing an AnaeroPack™‐Anaero Anaerobic Gas Generator (Thermo Fisher Scientific, Waltham, MA, USA) at 37 °C for 12 h, according to the manufacturer's instructions, to stimulate hypoxia. The oxygen concentration reached a level of < 1% within 1 h 16.

### Real‐time quantitative polymerase chain reaction (qPCR)

Total RNA was extracted from cells, followed by the synthesis of cDNA using reverse transcriptase and oligo‐dT primers. Subsequently, the cDNA samples were used as templates for real‐time PCR analysis, performed on an MX3005P™ instrument (Agilent Technologies, Santa Clara, CA, USA), using specific primers for each gene (Table 1). The expression levels of the genes examined were normalized using the *Actb* expression levels as an internal control for each sample 17.

**Table 1:** List of primers used for qPCR in this study

Genes	Upstream (5′‐3′)	Downstream (5′‐3′)
*Glut1*	GAGTGCCTGAAACCAGAGG	CTCACACTTGGGAGTCA
*Hif1a*	TCAAGTCAGCAACGTGGAAG	TATCGAGGCTGTGTCGACTG
*Hif2a*	GAGGAAGGAGAAATCCCGTGA	TATGTGTCCGAAGGAAGCTGA
*Hmox1*	CCAGACACCGCTCCTCCAG	GGATTTGGGGCTGCTGGTTTC
*Slc7a5*	ATATCACGCTGCTCAACGGTG	CTCCAGCATGTAGGCGTAGTC
*Slc7a8*	TGTGACTGAGGAACTTGTGGA	GTGGACAGGGCAACAGAAATG
*Slc43a1*	CTTCCGGGCTTCACCTATCTG	CCCAATTCCAAATCGCATCCAC
*Slc43a2*	TGCACCGCTGTGTTGGAAA	CCGTGCTGTTAGTGACATTCTC
*Actb*	AAACTGGAACGGTGAAGGCGAC	CAGAAGCAATGCTGTCACCTTCC

### Retroviral short hairpin RNA (shRNA) vector and infection

The retroviral shRNA vector for HIF‐1α was previously generated 18, and the shRNA vector for HIF‐2α was obtained from Sigma. Retroviral vectors were transfected into PLAT‐E cells using the calcium carbonate method. Virus supernatants were collected 48 h after transfection, and the cells were subsequently infected with virus supernatants for 24 h in the presence of 4 μg·mL^−1^ polybrene 19.

### Chromatin immunoprecipitation (ChIP) assay

ChIP experiments were performed according to the manufacturer's protocol 20. Briefly, Neuro2A cells were incubated with 1% formaldehyde at room temperature for 20 min. After centrifugation of the crosslinked samples, the pellet was homogenized and subjected to sonication using lysis buffer. Immunoprecipitation was performed using the anti‐HIF‐1α (#sc‐10790) (Santa Cruz, Dallas, TX, USA) and anti‐HIF‐2α (#ab199) (Abcam, Cambridge, UK) antibodies. DNA was extracted using phenol/chloroform, and PCR was performed using specific primers: (a) 5′‐ACTTGTGAGGAGGTGTGAGTCG‐3′ and (b) 5′‐TGTTGGTTCAGCTCGCAAAGG‐3′.

### Immunoblotting

Cultured cells were solubilized in lysis buffer containing 1% Nonidet P‐40 detergent. Samples were then subjected to SDS/PAGE, followed by transfer to polyvinylidene fluoride membranes and subsequent immunoblotting with anti‐β‐Actin (#sc‐47778) (Santa Cruz), anti‐p‐p70S6K1 (#9234), and anti‐p70S6K1 (#2708) (Cell Signaling Technology, Danvers, MA, USA).

### Data analysis

All results are expressed as the mean ± standard error of the mean, and statistical significance was determined using the two‐tailed, unpaired Student's *t*‐test, or two‐way analysis of variance using the Bonferroni *post hoc* test.

## Results and Discussion

**Figure 1:**
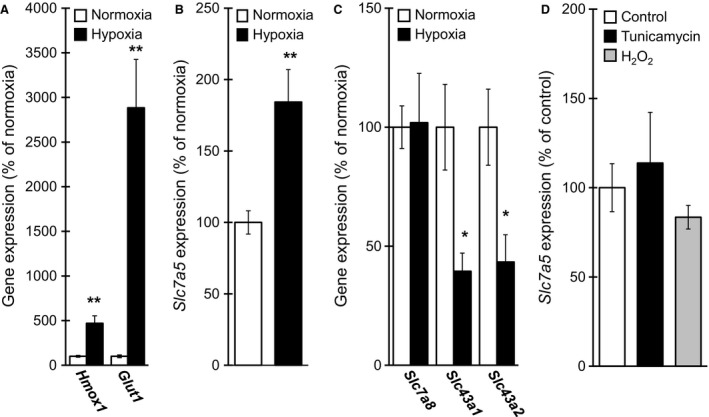
*Slc7a5* expression is upregulated by hypoxia in differentiated Neuro2A cells. Neuro2A cells were differentiated by ATRA and subsequently cultured for 12 h under hypoxia, followed by determination of mRNA expression of (A) *Hmox1* and *Glut1*, (B) *Slc7a5*, and (C) *Slc7a8*,* Slc43a1*, and *Slc43a2* by qPCR (*n* = 4). (D) Neuro2A cells were differentiated by ATRA and subsequently stimulated with tunicamycin or H_2_O_2_ for 12 h, followed by determination of mRNA expression of *Slc7a5* by qPCR (*n* = 4). *P < 0.05, **P < 0.01, significantly different from the value obtained in cells under normoxia. The data are expressed as the mean ± standard error of the mean; statistical significance was determined using the two‐tailed, unpaired Student's *t*‐test.

We initially determined the expression of *Slc7a5* in response to hypoxia in cultured neuronal cells. Differentiated Neuro2A cells were exposed to hypoxia for 12 h; further, the expression of *Slc7a5* was determined using qPCR. A marked upregulation in the expression of hypoxia‐responsive genes, such as heme oxygenase‐1 (*Hmox1*) and glucose transporter‐1 (*Glut1*) (Fig. 1A), was detected in these cells cultured under hypoxia for 12 h. Under this experimental condition, the expression of *Slc7a5* was significantly increased by hypoxia in differentiated Neuro2A cells (Fig. 1B). In addition to LAT1 (*Slc7a5*), three additional LATs have been identified, namely LAT2 (*Slc7a8*), LAT3 (*Slc43a1*), and LAT4 (*Slc43a2*) 21. No marked upregulation was observed in the expression of *Slc7a8* with hypoxia in differentiated Neuro2A cells, whereas significant decreases were observed in the expression of *Slc43a1* and *Slc43a2* (Fig. 1C). In contrast, neither endoplasmic reticulum (ER) stressor tunicamycin at 0.1 μg·mL^−1^ nor the oxidative stressor hydrogen peroxide (H_2_O_2_) at 100 μm significantly affected *Slc7a5* expression in differentiated Neuro2A cells (Fig. 1D). These results indicate that the expression of *Slc7a5* was selectively upregulated in differentiated Neuro2A cells by hypoxia but not by ER stressor and oxidative stressor.

**Figure 2:**
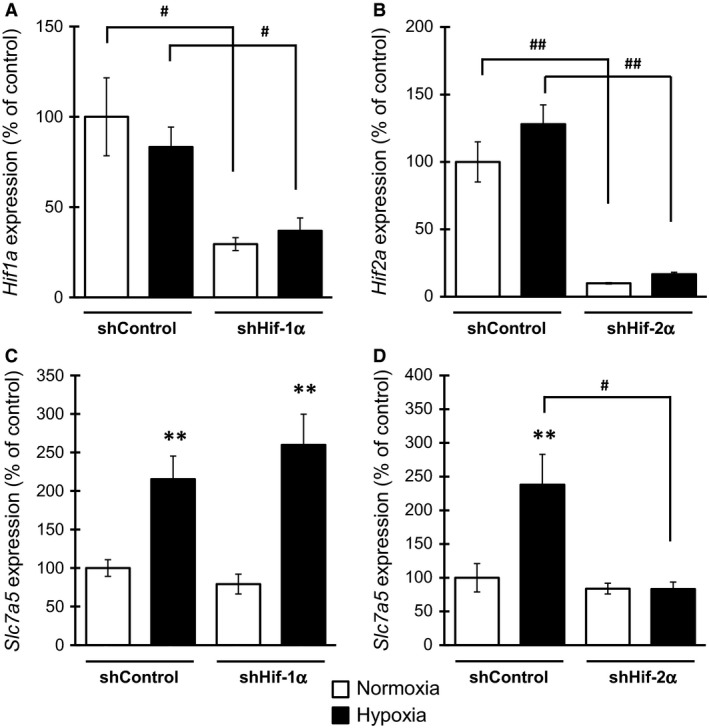
*Slc7a5* expression is upregulated by hypoxia through HIF‐2α in differentiated Neuro2A cells. Neuro2A cells retrovirally infected with shHif‐1α or shHif‐2α were differentiated by ATRA, followed by exposure to hypoxia and subsequent determination of (A) *Hif1a*, (B) *Hif2a*, and (C and D) *Slc7a5* expression. **P < 0.01, significantly different from the value obtained in cells under normoxia. ^#^ *P* < 0.05, ^##^ *P* < 0.01, significantly different from the value obtained in cells infected with shControl. The data are expressed as the mean ± standard error of the mean; statistical significance was determined using the two‐way analysis of variance using the Bonferroni *post hoc* test.

We subsequently investigated the regulation of hypoxia‐induced *Slc7a5* expression by HIF, a master transcriptional regulator of the adaptive response to hypoxia. Differentiated Neuro2A cells were retrovirally infected using shRNA for HIF‐1α (shHif‐1α) or HIF‐2α (shHif‐2α), followed by culture under hypoxia for 12 h and subsequent determination of the expression of *Slc7a5*. We initially confirmed that the expression of *Hif1a* or *Hif2a* was significantly decreased in differentiated Neuro2A cells through retroviral infection using shHif‐1α or shHif‐2α, respectively, under both normoxia and hypoxia, without alteration of their expression by hypoxia in cells infected with shControl, shHif‐1α, or shHif‐2α (Fig. 2A,B). Under these experimental conditions, the expression of *Slc7a5* was markedly increased by hypoxia in cells transfected with shControl. Additionally, a significant upregulation of *Slc7a5* expression under hypoxia was observed in cells transfected with shHif‐1α but not with shHif‐2α. Moreover, the hypoxia‐induced upregulation of the expression of *Slc7a5* was completely blocked through infection with shHif‐2α, but not with shHif‐1α (Fig. 2C,D). Collectively, the hypoxia‐induced expression of *Slc7a5* was regulated by the transcription factor HIF‐2α in differentiated neuronal cells.

**Figure 3:**
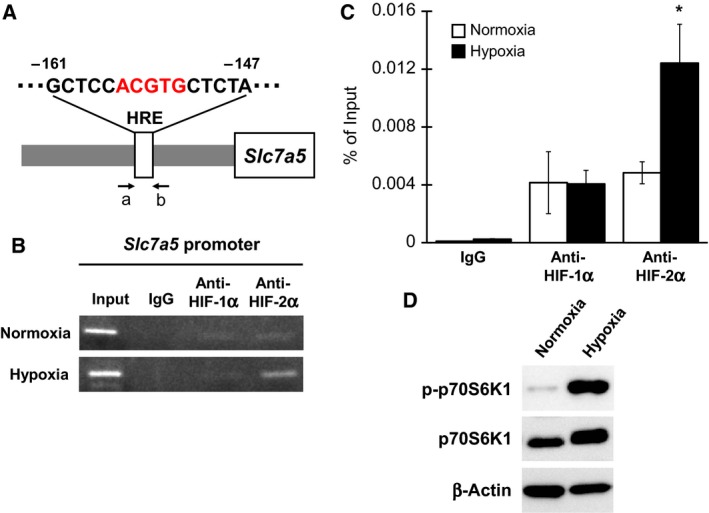
Hypoxia increases the recruitment of HIF‐2α on *Slc7a5* proximal promoter in differentiated Neuro2A cells. (A) Schematic representation of the *Slc7a5* promoter region with putative HIF binding site in addition to primer regions for ChIP assay. (B and C) Neuro2A cells were differentiated by ATRA and subsequently cultured under hypoxia, followed by ChIP assays using the anti‐HIF‐1α or anti‐HIF‐2α antibody along with specific primers to amplify the *Slc7a5* promoter containing HRE. (D) Neuro2A cells were differentiated by ATRA and subsequently cultured under hypoxia for 12 h, followed by immunoblotting (*n* = 3). *P < 0.05, significantly different from the value obtained in cells under normoxia. The data are expressed as the mean ± standard error of the mean; statistical significance was determined using the two‐tailed, unpaired Student's *t*‐test.

Computational analysis of the 5′‐flanking region of the highly conserved mouse and human *Slc7a5* genes identified one putative HIF‐responsive element (HRE) (Fig. 3A). Subsequently, we investigated the role of HIF recruitment to the promoter region of the *Slc7a5* gene in the upregulation of the expression of *Slc7a5* in differentiated Neuro2A cells under hypoxic conditions. The cells were exposed to hypoxia for 12 h, followed by immunoprecipitation using the anti‐HIF‐1α or anti‐HIF‐2α antibodies and PCR using primers (primers a and b indicated in Fig. 3A) to amplify a region containing the HRE of the *Slc7a5* promoter. The recruitment of HIF‐1α to the *Slc7a5* promoter region was equally observed in differentiated Neuro2A cells under normoxia and hypoxia (Fig. 3B,C). Alternatively, the recruitment of HIF‐2α to the *Slc7a5* promoter region was markedly increased through exposure to hypoxia in differentiated Neuro2A cells (Fig. 3B,C). These results indicate that HIF‐2α binds directly to the putative binding site located at the *Slc7a5* promoter, leading to the upregulation of the expression of *Slc7a5* under hypoxia in differentiated neuronal cells.

It has been previously shown that the activation of the HIF‐2α pathway was reported to increase mTORC1 activity by upregulating expression of *Slc7a5* in tumor cells and in lung tissue 22. Accordingly, the finding that hypoxia induces *Slc7a5* expression through HIF‐2α in this study led us to examine whether hypoxia can increase mTORC1 activity in differentiated neuronal cells. The phosphorylated form of p70S6K1, a downstream marker of mTORC1, was markedly increased through exposure to hypoxia for 12 h in differentiated Neuro2A cells (Fig. 3D), indicating that hypoxia induces not only *Slc7a5* expression but also mTORC1 activation in differentiated neuronal cells.

Maintaining normal levels of BCAAs in the brain is critical for brain homeostasis. Abnormal levels of BCAAs lead to pathophysiological implications such as ASD. In addition to the uptake of BCAAs, LAT1 has been implicated in the delivery of pharmaceutical drugs into the brain, including L‐DOPA and gabapentin, and thyroid hormones 23, 24, 25. Therefore, LAT1 (*Slc7a5*), expressed at the surface of neuronal cells, may play a crucial role in drug delivery and hormonal input into the brain, in addition to amino acid sensing.

It should be noted that hypoxia decreased mRNA expression of *Slc43a1* and *Slc43a2*, which do not contain conserved HRE between mouse and human, in differentiated Neuro2A cells. Although the exact mechanism underlying the downregulation of *Slc43a1* and *Slc43a2* should be elucidated, in this study, we focused on the regulatory mechanism by which hypoxia/HIF axis controls the expression of *Slc7a5*.

In this study, we demonstrated that the amino acid transporter gene *Slc7a5* may be a novel gene responsive to hypoxia in a HIF‐2α‐dependent manner in differentiated neuronal cells. *Slc7a5* global knockout in mice is embryonically lethal at E9.5–10.5. Therefore, the *in vivo* vital role of *Slc7a5* expressed by neurons in the development, homeostasis, and pathology of the CNS remains highly unclear 26. mTORC1 is implicated in neurodegenerative and neuropsychiatric diseases as well as in various physiological brain functions, including learning, memory, and synaptic plasticity 27. Additional studies are warranted to determine the crucial role of upregulated *Slc7a5* in neuronal functions by hypoxic condition. However, the fact that hypoxia increased both *Slc7a5* expression through HIF‐2α and mTORC1 activity in differentiated neuronal cells suggests one possible but hitherto unidentified speculation that hypoxia/HIF‐2α/LAT1 axis in neurons can be implicated in the modulation of mTORC1‐dependent neuronal function under physiological and pathological conditions. In any case, LAT1 (*Slc7a5*) expressed in neurons may be a target for the discovery and development of novel therapeutic agents against various neurodegenerative and neuropsychiatric diseases.

## Conflict of interest

The authors declare no conflict of interest.

## Author contributions

YO and EH conceived and designed the study. YO, MH, HK, TI, and TY performed experiments. KK and EH discussed the results and provided comments. YO and EH wrote the manuscript.
